# Oct4 Mediates Tumor Initiating Properties in Oral Squamous Cell Carcinomas through the Regulation of Epithelial-Mesenchymal Transition

**DOI:** 10.1371/journal.pone.0087207

**Published:** 2014-01-27

**Authors:** Lo-Lin Tsai, Fang-Wei Hu, Shiuan-Shinn Lee, Chuan-Hang Yu, Cheng-Chia Yu, Yu-Chao Chang

**Affiliations:** 1 School of Dentistry, Chung Shan Medical University, Taichung, Taiwan; 2 Department of Dentistry, Chung Shan Medical University Hospital, Taichung, Taiwan; 3 School of Public Health, Chung Shan Medical University, Taichung, Taiwan; 4 Institute of Oral Sciences, Chung Shan Medical University, Taichung, Taiwan; University of Alabama at Birmingham, United States of America

## Abstract

**Background:**

Overexpression of Oct4, an important transcription factor of embryonic stem cells (ESC), has been reported in several cancers. The aim of this study was to determine the emerging role of Oct4 in oral squamous cell carcinoma (OSCC) both *in vitro* and *in vivo*.

**Methodology/Principal Finding:**

Tumourigenic activity and molecular mechanisms of Oct4 overexpression or knockdown by lentiviral infection in OSCC was investigated *in vitro* and *in vivo*. Initially, we demonstrated that Oct4 expression was increased in OSCC cell lines as compared to a normal oral epithelial cell line SG. Overexpression of Oct4 was demonstrated to enhance cell proliferation, invasiveness, anchorage-independent growth and xenotransplantation tumourigenicity. These findings were coupled with epithelial-mesenchymal transition (EMT) transformation in OSCCs. In contrast, the silence of Oct4 significantly blocked the xenograft tumorigenesis of OSCC-derived cancer stem cells (OSCC-CSCs) and significantly improved the recipient survival. Clinically, the level of Oct4 expression was higher in recurrent and metastatic OSCC specimens but lower in primary OSCC specimens.

**Conclusion/Significance:**

Our results suggest that Oct4-mediated tumorigenecity is associated with the regulation of EMT. Oct4 might be a therapeutic target for OSCC.

## Introduction

Oral squamous cell carcinoma (OSCC) is the sixth most prevalent malignancy worldwide and accounts for approximately 8–10% of all cancers in Southeast Asia [1]. The prognosis of OSCC remains dismal because more than 50% of patients die from this disease or complications within 5 years with current therapies [2]. Therefore, an improved comprehension of the cellular and molecular mechanisms which initiate tumorigenesis or promote cancer progression, was pursued to bring forth future progress of medical treatment of OSCC.

Oct4 is the key transcription factor that is involved in the maintenance of pluripotency and self-renewal in undifferentiated embryonic stem (ES) cells [3]. Oct4 could reprogram human somatic fibroblasts into embryonic stem cell-like pluripotent cells which are termed as inducible pluripotent stem cells (iPSC) [4]. Oct4 has been reported to be overexpressed in various cancers including germ cell tumors [5],breast [6], cervix [7], oral [8], prostate [9], lung [10], gastric [11], brain [12], liver [13], and ovarian cancer [14]. Oct4 is also to be a key determinant of cancer stem cells (CSCs) properties [15]. Previously, we have demonstrated that Oct4 is upregulated in OSCC-CSCs and OSCC specimens are negatively correlated with the survival rate of OSCC patients [8]. However, Oct4 mediated molecular mechanisms in OSCC still remain to be elucidated.

Epithelial mesenchymal transition (EMT) is a de-differentiation program that converts adherent epithelial cells into individual migratory cells. EMT is thought to be a key step in the induction of tumor malignancy, oncogenic progression, and cancer metastasis [16]. Recently, the interplay between EMT and a cancer stemness signature has gained huge interest in the field of cancer research [17]. Researchers have shown that EMT can promote stemness properties and further generate cells with the features of breast CSCs [18]. The EMT program is also reported to play an important role to maintain the property of tumor initiating cells in OSCCs [19]. The detailed molecular mechanisms involved in the regulatory links between Oct4 and EMT properties are still poorly understood. Herein, we demonstrate a critical role of Oct4 overexpression in the enhancement of stemness and EMT in OSCCs. In addition, an animal model revealed that the inhibition of Oct4 may improve the outcome of OSCCs.

## Materials and Methods

### OSCC tissues acquirement and preparation

Surgical tissue specimens from OSCC patients were collected after obtaining written informed consent and this study was approved by The Institutional Review Board in Chung Shan Medical University Hospital (CSMUH No: CS10249). Human primary OSCC carcinoma (T) tissue, normal paired noncancerous matched tissues (N), as well as available lymph node metastatic lesions (M) were obtained from surgical procedures sent to the pathology lab for frozen section diagnosis. Tumor tissues were microscopically screened to have >70% of their areas occupied by tumor cells; The remaining specimen (tumor, normal counterpart, and lymph node metastatic lesions) were snap frozen in liquid nitrogen and stored at −80°C for Real-time Reverse transcription–PCR (qRT-PCR).

### Cell lines

Nine OSCC cell lines (SAS [20], FaDu [21], OECM1 [22], SCC4 [23], HSC3 [24], OC3 [25], Ca922 [26], SCC25 [27], and GNM [28] and one normal gingival epithelial cell line SG [29] were used in this study.

### SYBR real-time reverse transcription-polymerase chain reaction (RT-PCR)

Total RNA of cells was purified using Trizol reagent (Invitrogen, Carlsbad, CA, USA) according to the manufacturer's protocol. Briefly, the total RNA (1 µg) of each sample was reversely transcribed by Superscript II RT (Invitrogen, Carlsbad, CA, USA). Then, the amplification was carried out in a total volume of 20 µl containing 0.5 µM of each primer, 4 mM MgCl_2_, 2 µl LightCycler™ –FastStart DNA Master SYBR green I (Roche Molecular Systems, Alameda, CA, USA) and 2 µl of 1:10 diluted cDNA. The *GAPDH* housekeeping gene was amplified as a reference standard. *GAPDH* primers were designed: *GAPDH* (forward): GGGCCAAAAGGGTCATCATC (nt 414–434, GenBank accession no. BC059110.1), *GAPDH* (reverse): ATGACCTTGCCCACAGCCTT (nt 713–733). PCR reactions were prepared in duplicate and heated to 95°C for 10 minutes followed by 40 cycles of denaturation at 95°C for 10 seconds, annealing at 55°C for 5 seconds, and extension at 72°C for 20 seconds. All PCR reactions were performed in triplicate. Standard curves (cycle threshold values versus template concentration) were prepared for each target gene and for the endogenous reference (*GAPDH*) in each sample. To confirm the specificity of the PCR reaction, PCR products were electrophoresed on a 1.2% agrose gel [30]. Primer sequences are listed in Table 1.

**Table 1:** The sequences of the primers for quantitative RT-PCR.

Gene (Accession No.)	Primer Sequence (5′ to 3′)	Product size (bp)	Tm (°C)
Slug (NM_003068)	F: GTGATTATTTCCCCGTATCTCTAT, R: CAATGGCATGGGGGTCTGAAAG	292	50
E-caderin (NM_004360)	F: ATTCTGATTCTGCTGCTCTTG, R: AGTCCTGGTCCTCTTCTCC	136	50
N-caderin (NM_001792)	F: CCACGCCGAGCCCCAGTATC, R: CCCCCAGTCGTTCAGGTAATCA	232	55
Oct-4 (NM_002701)	F: GTGGAGAGCAACTCCGATG, R: TGCTCCAGCTTCTCCTTCTC	86	60
GAPDH (NM_002046)	F: CATCATCCCTGCCTCTACTG, R: GCCTGCTTCACCACCTTC	180	60

### Cell proliferation assay

An MTT assay kit (Sigma-Aldrich) was used to analyze the cell proliferation. Specifically, 1×10^3^ cells were seeded in each well of a 24-well plate, and then 10 µL of MTT solution was added to the cells which were then incubated at 37°C for 3 hours. The supernatant was removed, and 200 µL of DMSO were added directly to the cells. The MTT color reaction was analyzed using a microplate reader set at *A*560 nm.

### Stable overexpression of Oct4 in OSCC cells

Human full-length Oct4 cDNA was cloned into pCDH1-MCS1-EF1-copGFP (System Biosciences, Cat. No: CD511A-1; Mountain View, CA, USA). Lentivirus production was performed by co-transfection of a plasmid DNA mixture with lentivector plus helper plasmids (VSVG and Gag-Pol) into 293 T cells (American Type Culture Collection, Manassas, VA, USA) using Lipofectamine 2000 (LF2000, Invitrogen, Calsbad, CA, USA). The lentivirus M.O.I titer was determined by flow cytometry (average of 5×10^4^ and 2×10^5^ TU/ml). To generate the stable cell lines, sub-confluent OSCC cells were infected with lentivirus in the presence of 8 µg/ml polybrene (Sigma-Aldrich, St Louis, MO, USA). Stable Oct4-overexpressing OSCC cells were further purified by cell sorting with GFP positive cells. The pCDH1-MCS1-EF1-copGFP empty vector alone was utilized for the experimental control [31].

### Construction of lentiviral-mediated RNAi for silencing Oct4

The pLV-RNAi vector, which co-expressed GFP protein in infected host cells, was purchased from Biosettia Inc. (Biosettia, San Diego, CA, USA). The method of cloning the double-stranded shRNA sequence was described in the manufacturer's protocol. Lentiviral vectors expressing shRNA that targets human Oct4 were synthesized and cloned into pLVRNAi to generate a lentiviral expression vector. Sh-Luc:5′-CCGGACTTACGCTGAGTACTTCGAACTCGAGTTCGAAGTACTCAGCGTAAGTTTTTTG-3′ was utilized for an experimental control. Lentivirus production was performed as above. Stable pLV-RNAi expressed OSCC cell lines were further purified by cell sorting with GFP positive cells [31].

### *In vitro* cell invasion assay

For transwell migration assays, 2×10^5^ cells were plated into the top chamber of a transwell (Corning, Acton, MA, USA) with a porous membrane (8.0 µm pore size). Cells were plated in medium with lower serum (0.5% FBS) and medium supplemented with higher serum (10% FBS) was used as a chemoattractant in the lower chamber. The cells were incubated for 24 h at 37°C and cells that did not migrate through the pores were removed by a cotton swab. Cells on the lower surface of the membrane were stained with Hoechst 33258 (Sigma-Aldrich Co., St. Louis, MO, USA) to show the nuclei; fluorescence was detected at a magnification of 100× using a fluorescence microscope (Carl Zeiss, Oberkochen, Germany). The number of fluorescent cells in a total of five randomly selected fields was counted. In vitro cell invasion analysis was conducted as described previously [8].

### Tumorsphere-forming assay

Tumor cells were dissociated and cultured as tumorspheres in modified DMEM/F-12 supplemented with N2 (Invitrogen, Carlsbad, CA, USA), 10 ng/mL epidermal growth factor (EGF, Invitrogen, Carlsbad, CA, USA), 10 ng/mL basic fibroblast growth factor (bFGF, Invitrogen, Carlsbad, CA, USA), and penicillin/streptomycin at 10^3^ live cells/low-attachment six-well plate (Corning Inc., Corning, NY, USA), and the medium was changed every other day until the tumor sphere formation was observed in about 2 weeks. For serial passage of spheroid cells, single cells were obtained from accurtase-treated spheroids and the cell density of passage was 1000 cells/ml in the serum-free medium as described above [30].

### Soft agar colony forming assay

Each well (35 mm) of a six-well culture dish was coated with 2 ml of bottom agar (Sigma-Aldrich Co., St. Louis, MO, USA) mixture (DMEM, 10% (v/v) FCS, 0.6% (w/v) agar). After the bottom layer was solidified, 2 ml of top agar-medium mixture (DMEM, 10% (v/v) FCS, 0.3% (w/v) agar) containing 2×10^4^ cells was added, and the dishes were incubated at 37°C for 4 weeks. Plates were stained with 0.005% Crystal Violet, then the colonies were counted. The total number of colonies with a diameter ≥100 µm was counted over five fields per well for a total of 15 fields in triplicate experiments [30].

### Subcutaneous xenografts in nude mice

All the animal practices in this study were approved and in accordance with the Institutional Animal Care and Use Committee (IACUC) of Chung Shan Medical University, Taichung, Taiwan. 1×10^6^ OSCC cells mixed with Matrigel (BD bioscience, San Diego, CA, USA) (1:1) were injected subcutaneously into BALB/c nude mice (6–8 weeks). Tumor volume (TV) was calculated using the following formula: TV (mm^3^)  =  (Length × Width ^2^)/2 [32].

### Statistical analysis

A Statistical Package of Social Sciences software (version 13.0) (SPSS, Inc., Chicago, IL) was used for statistical analysis. Student's *t* test was used to determine statistical significance of the differences between experimental and control groups; *p* values less than 0.05 were considered statistically significant.

## Results

### Expression of Oct4 in OSCC cell lines

**Figure 1:**
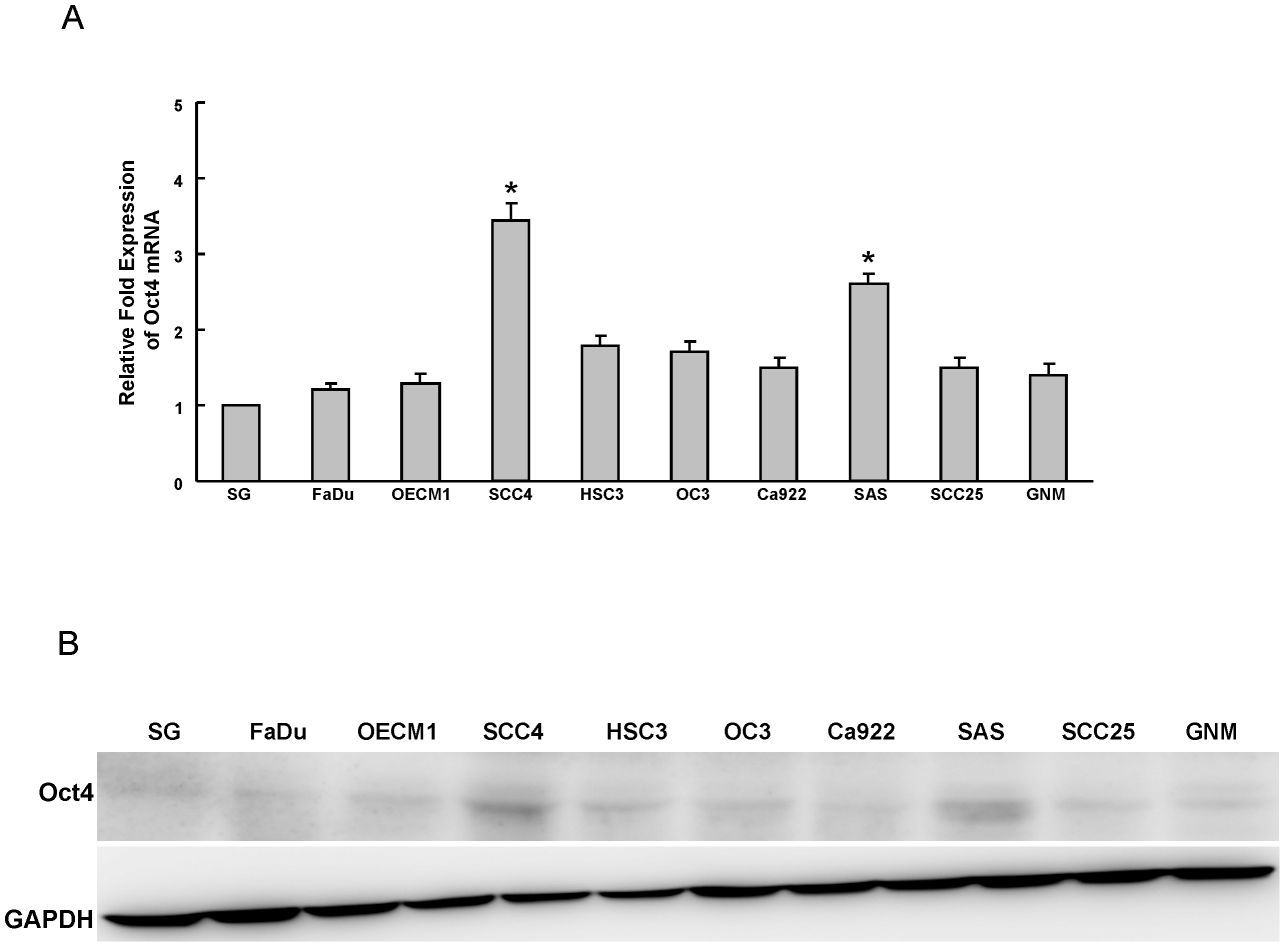
Determination of Oct4 expression in OSCC cells. Oct4 mRNA (A) and protein (B) expression in nine OSCC lines and one normal oral epithelial cell (SG) were examined by real-time RT-PCR analysis and western blotting. The amount of GAPDH protein of different crude cell extracts was referred to as a loading control. Error bars correspond to SD. (*, p<0.05)

To understand the expression of Oct4 in OSCC cell lines (OSCCs), the endogenous protein level of Oct4 in nine established OSCC cell lines and one normal oral epithelial cell line SG was examined by real-time RT-PCR and western blot analyses. As shown in figure 1A and 1B, Oct4 mRNA and protein were detectable in OSCC cell lines SSC4 and SAS OSCCs. However, it was lower or undetectable in normal oral epithelial cell line SG (Fig. 1A and B).

### Oct4 overexpression enhanced cell proliferation, invasiveness, and colony formation

**Figure 2:**
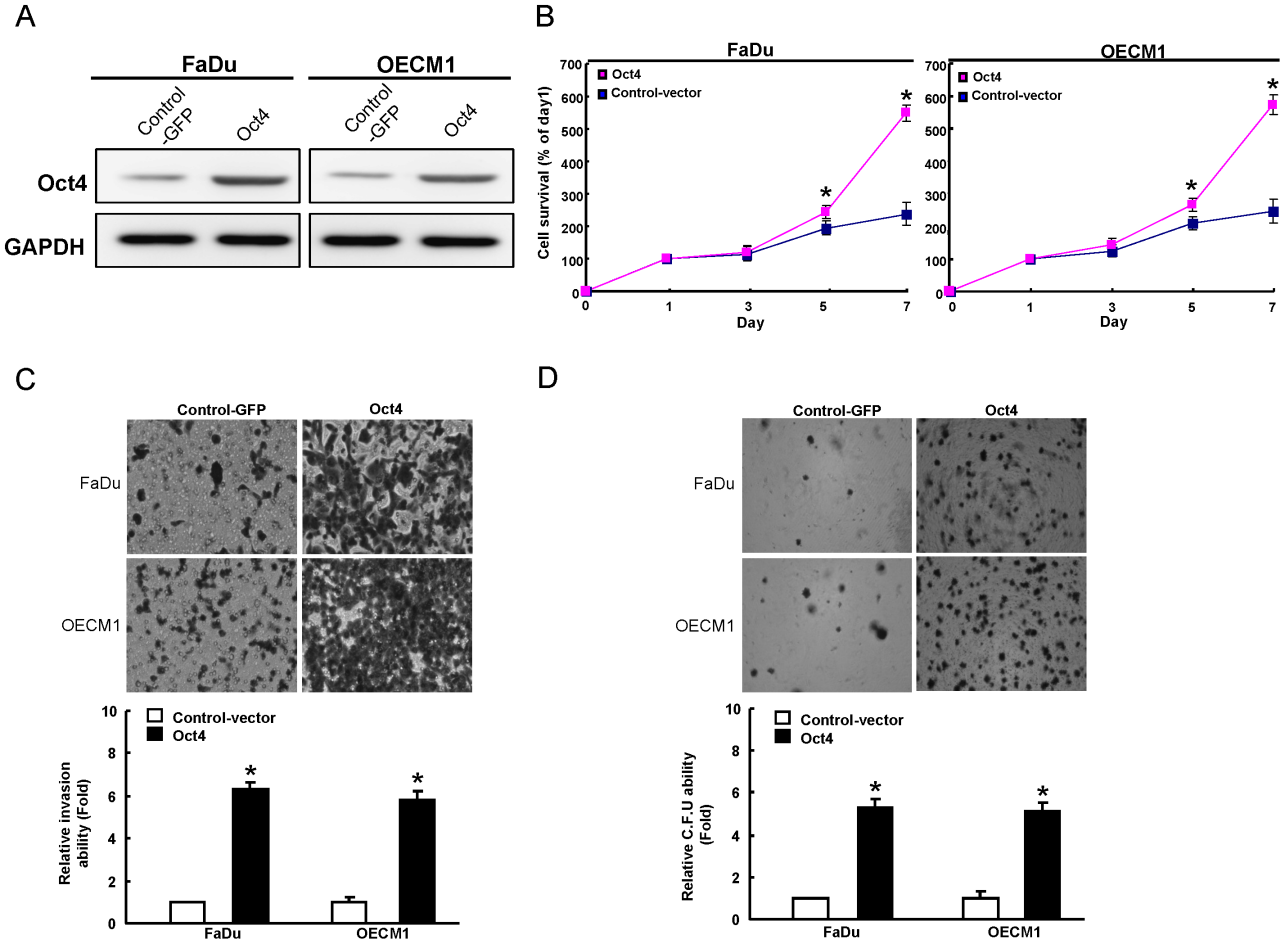
Overexpression of Oct4 in OSCCs enhances *in vitro* tumorigenic properties. (A) Total proteins were prepared from control vector–expressing and Oct4-overexpressing OSCCs (OECM1 and FaDu) and analyzed by immunoblotting against anti-Oct4 or anti-GAPDH antibodies as indicated. The amount of GAPDH protein of different crude cell extracts was referred to as a loading control. OECM1 and FaDu cells were transfected with Oct4 or control vector DNA and examined for (B) proliferation ability, (C) Matrigel invasion ability, and (D) anchorage-independent growth as described in the Materials and Methods section. The data shown are the means ± SD from three independent experiments.

To further investigate the upregulation of Oct4 on the biological properties of OSCC cell lines, we generated stable Oct4-overexpressing OSCC cell lines through lentiviral-mediated transduction. As shown in figure 2A, two Oct4-overexpressing OSCC cell lines, FaDu and OECM1, displayed elevated Oct4 expression by western blot analysis. Oct4-overexpressing OSCC cell lines showed enhanced proliferative activity (Fig. 2B). In addition, Oct4-overexpressing OSCC cell lines also resulted in increased ability of cell invasiveness (Fig. 2C) and colony formation (Fig. 2D). Collectively, these results suggest that overexpression of Oct4 may promote in vitro tumorigenicity of OSCC cells.

### Oct4 overexpression enhances *in vivo* tumourigenicity and mesenchymal traits in OSCCs

**Figure 3:**
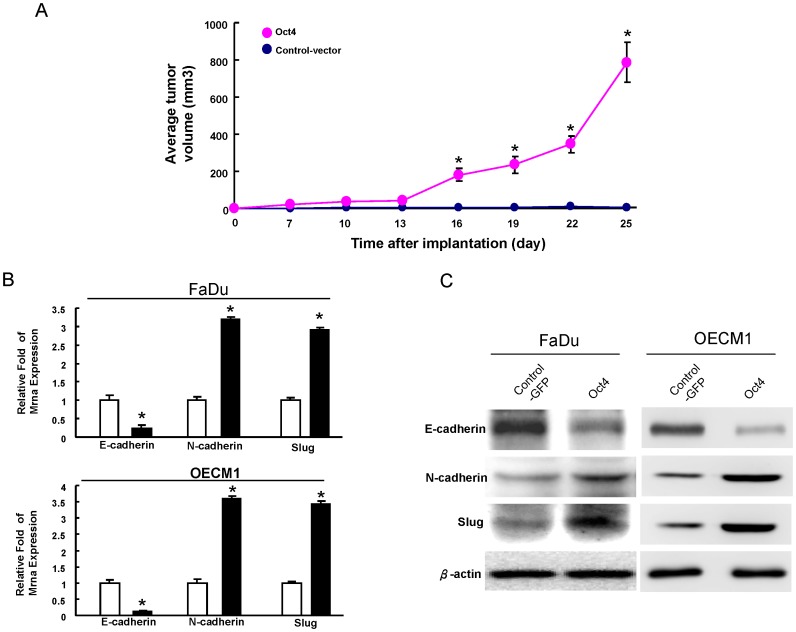
Overexpression of Oct4 in OSCCs promotes in vivo tumorigenicity and EMT properties. (A) FaDu cells were transfected with Oct4 or control vector. The transfected cells were injected subcutaneously into the flank of nude mice. FaDu cells were transfected with or control vector and Oct4. Cell extracts from the transfected cells were analyzed for the EMT marker by real-time RT-PCR analysis (B) and western blot (C).

The comparison between GFP expressing control cells and Oct4-overexpressing OSCC cell lines showed a significant increase in tumorigenic ability of OSCC cells *in vivo* (Fig. 3A). Previously, Yang et al. (2008) reported that OSCC cells expressed with elevated EMT markers were highly metastatic, tumorigenic, and resistant to radiotherapy/chemotherapy [33]. As shown in figures 3B and 3C, we also found the reduced expression of an epithelial marker, E-cadherin, but the enhanced expression of mesenchymal markers N-cadherin and Slug in Oct4-overexpressing OSCCs.

### Targeting Oct4 effectively abrogates *in vitro* and *in vivo* malignant properties of OSCC-CSCs

**Figure 4:**
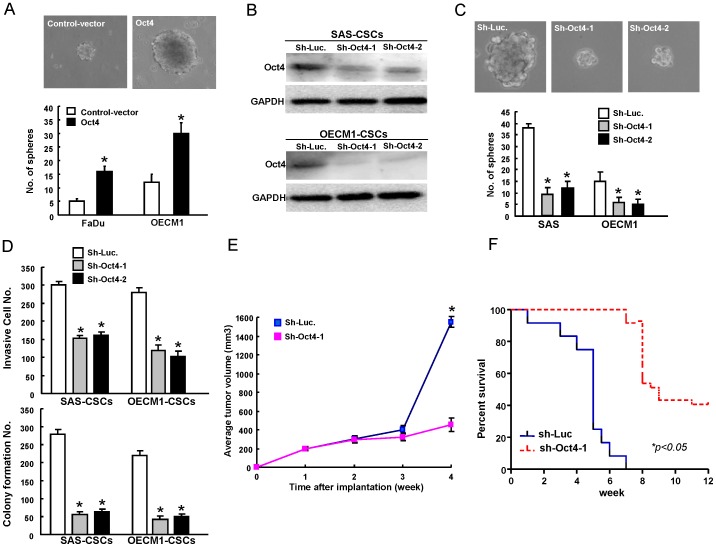
Oct4 downregulation abrogate the cancer stemness in OSCC-CSCs. (A) Representative images of tumor sphere formation ability of control-GFP or Oct4-overexpressing OSCCs. (B) Single cell suspensions of OSCC-CSCs were transduced with sh-Luc or sh-Oct4 lentivirus for 3 days. Total proteins prepared from infected cells were prepared and analyzed. The silencing effect of Oct4 shRNA in OSCC-CSCs was validated translationally by western blotting (OECM1 (*left panel*) and SAS (*right panel*)). Immunoblotting against anti-Oct-4 or anti-GAPDH antibodies was performed as indicated. The amount of GAPDH protein of different crude cell extracts was referred to as a loading control, and for further quantification. (C) OSCC-CSCs were first infected with sh-Oct-4-1, sh-Oct-4 -2 or sh-Luc lentivirus for 3 days, and then further cultivated under the serum-free defined selection medium. The cellular morphology of OSCC-CSCs treated with sh-Luc or Oct-4-shRNA lentivirus was examined. (D) To elucidate the capability of cell invasiveness (*upper panel*) and anchorage independent growth (*lower panel*) of OSCC-CSCs, OSCC-CSCs with Oct-4 down-regulation, single cell suspension of OSCC-CSCs infected with Oct-4-specific shRNA or control sh-Luc lentivirus for three days were plated onto transwells coated with matrigel and soft agar, respectively, and analyzed as described in the Materials and Methods section. Results are means ± SD of triplicate samples from three experiments. (E) Representative tumors of control and Oct4-knockdown OSCC-CSCs were generated, and the tumors were then dissected from the subcutaneous space of recipient mice (n = 3). (F) Kaplan–Meier survival analysis further indicated the mean survival rates of animals transplanted with control and Oct4-knockdown OSCC-CSCs.

Sphere-forming OSCC cells have been shown to exhibit cancer stem cells properties [8], [30]. In this study, we displayed the up-regulation of Oct4 in OSCC-CSCs from FaDu and OECM1 cells. Oct4-overexpressing OSCCs could enhance the sphere-forming ability (Fig. 4A). To further investigate whether Oct4 could play a role in maintaining CSCs properties of OSCC-CSC, the measurement of loss-of-function of Oct4 was first conducted. Down-regulation of Oct4 in OSCCs-CSCs was achieved by viral transduction with lentiviral vector expressing small hairpin RNA (shRNA) targeting Oct4 (sh- Oct4-1 and sh-Oct4-2). Lentiviral vector expressing shRNA against luciferase (sh-Luc) was used as the control. Immunoblotting analyses confirmed that lentivirus expressing both sh-Oct4-1 and sh- Oct4-2 markedly reduced the expression level of Oct4 protein in transduced OSCCs-CSCs (Fig. 4B). Down-regulation of Oct4 decreased the self-renewal capacity of OSCCs-CSCs (Fig. 4C). Infection with sh- Oct4 expressing lentivirus significantly decreased matrigel invasion and anchorage independent growth of OSCCs-CSCs (Fig. 4D). The inhibition of Oct4 expression significantly slowed down the tumor growth mediated by OSCC-CSC (Fig. 4E). Additionally, the survival of OSCCs-CSCs-transplanted mice was significantly improved upon Oct4 knockdown treatment (Fig. 4F). Taken together, these data further support the conclusion that the down-regulation of Oct4 may result in the reduction of CSCs properties in OSCCs.

### The upregulation of Oct4 expression in recurrent and metastatic OSCC patients

**Figure 5:**
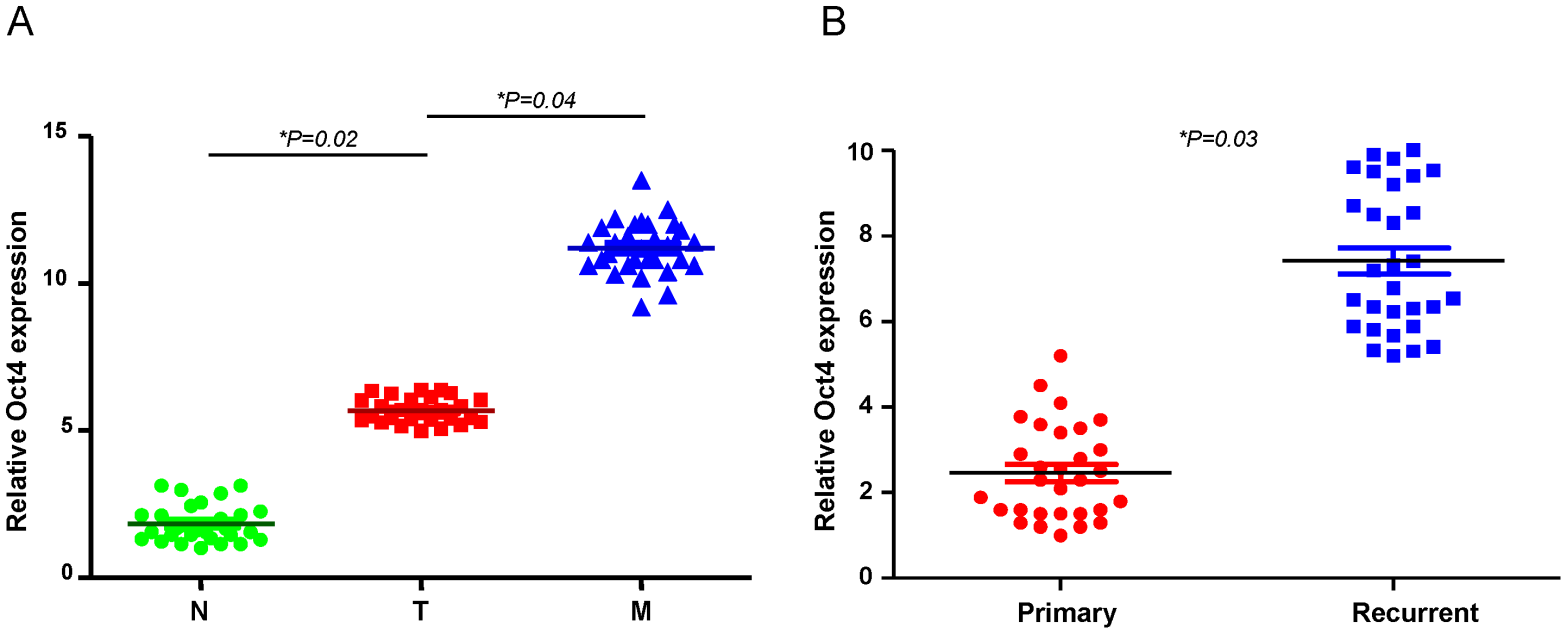
Clinical significance of Oct4 expression in recurrent and metastatic OSCC patients. (A) Quantitative RT-PCR analysis of Oct4 expression levels in clinical specimens from a non-tumor region (N) or a tumor region (T) or lymph node (N) of OSCC patients with metastases. (J) (B) Quantitative RT-PCR analysis of Oct4 expression levels in clinical specimens from primary (P) and recurrent (R) OSCC patients.

To validate the significance of Oct4 expression in clinical specimens, we collected paired samples of non-tumor (N), local tumor (T), and lymph node (LN) tissues from OSCC patients and subjected these samples to real-time RT-PCR analysis. Compared with non-tumor samples from the same patient, the expression of Oct4 was increased in all of the tumor samples (Fig. 5A). A similar upregulation of Oct4 was also observed in metastatic lymph nodes when compared with local tumors (T) (Fig. 5A). We also compared the levels of these molecules between primary and recurrent lesions in OSCC patient tissues. In line with our previous data, the level of Oct4 expression was higher in recurrent OSCC tumor samples but lower in primary lesions (Fig. 5B). These findings revealed a high Oct4 expression signature as a potential marker of metastasis and recurrence of OSCC.

## Discussion

OSCC is one of the leading causes of cancer-related deaths worldwide [34]. Its high invasiveness and frequent recurrence are the major reasons for treatment failures and a poor prognosis [2]. Accumulating data demonstrate that a CSCs hypothesis has been bolstered by the clinical observation that malignant tumors are relatively resistant to chemo-radiotherapies [35], [36]. OSCC-derived CSCs have been known to have the capacity to promote tumor progression and metastasis, and also contribute to radio-resistance and chemo-resistance [37], [38]. Thus, it is necessary to unravel the underlying mechanisms of the CSC pathway in OSCC and to further evaluate the therapeutic possibilities of CSC-targeted therapy clinically. It is generally accepted that several embryonic stem cell-specific signalings are reactivated in CSC by several cancer models [39]–[41]. The embryonic stem cell-specific transcriptional factor Oct4 was shown to be highly expressed in hepatic, colorectal, and brain CSCs [12], [13], [39]. Our previous report demonstrated high expression of Oct4 in malignancies of OSCC-CSCs and OSCC patients [8]. Chiou *et al.* also demonstrated the overexpression of Oct4 and Nanog transformed lung cancer cells into a CSCs-like state [42]. In the present study, we directly evaluated the role of Oct4 in the maintenance of tumorigenic potential of OSCCs by lentiviral shRNAi mediated overexpression and lentiviral-mediated knockdown of Oct4. Overexpression of Oct4 enhanced tumorigenic properties of OSCCs both in vitro and in vivo (Fig. 2& Fig. 3). Depletion of Oct4 decreased the sphere-forming capability, invasion ability, and xenograft tumorigenesis of OSCC-CSCs and largely improved recipient survival (Fig. 4). These results revealed that Oct4 could promote OSCC malignancy. Therefore, Oct4 may serve as a therapeutic target for the treatment of metastatic OSCC.

EMT is critical for embryonic development and oncogenic progression [16]. Yang *et al.* found that both Twist1 and Bmi-1 were mutually essential to promote EMT and tumor-initiating capability [43]. Enhanced EMT characteristics are associated with poor overall and metastasis-free survival in patients with OSCC [19], [33]. Recent studies suggested that EMT promotes stemness properties in normal breast tissue and breast cancer cells [18], [44]. Results support the idea that Oct4/Nanog signaling regulates tumor-initiating abilities, and promotes metastasis in lung cancer cells through regulation of EMT [42]. Slug Snail, and Twist, EMT-related transcription factors, have been reported to increase the metastatic ability and cancer stemness in cancer cells [45]-[47]. To the best of our knowledge, we are the first to report that Oct4 could regulate tumor initiating property and EMT traits, at least in part (Fig. 3). Overexpression of Oct4 in OSCC cells resulted in an EMT-favored pattern in which mesenchymal markers such as Slug and N-cadherin were increased (Fig. 3C). The discovered roles of Oct4 coupled with the EMT process has stimulated a huge interest in the field of cancer research as it indicates that misplaced cancer stemness properties contribute to tumor metastasis and recurrence, making cancer difficult to treat. A further understanding on the regulatory networks between EMT and stemness signatures may update our current knowledge on the development of therapeutic treatments for malignant cancers in the future.

It is of interest to further elucidate the mechanisms by which downstream factors regulated by Oct4 expression in OSCC *in vivo* and to understand how Oct4 mediates EMT and cancer stemness in OSCC. Overall, targeting Oct4 in malignant OSCC may create a new approach for therapeutic treatments in the future.
